# Down-Regulation of Filamin Ainteracting protein 1-like Is Associated with Promoter Methylation and an Invasive Phenotype in Breast, Colon, Lung and Pancreatic Cancers

**DOI:** 10.1371/journal.pone.0082620

**Published:** 2013-12-05

**Authors:** Mijung Kwon, Soo Jin Lee, Srilakshmi Reddy, Yevangelina Rybak, Asha Adem, Steven K. Libutti

**Affiliations:** Department of Surgery, Albert Einstein College of Medicine of Yeshiva University, Bronx, New York, United States of America; Wayne State University, United States of America

## Abstract

Identifying key mediators of cancer cell invasion and metastasis is critical to the development of more effective cancer therapies. We previously identified Filamin A interacting protein 1-like (FILIP1L) as an important inhibitor of cell migration and invasion in ovarian cancer. FILIP1L expression was inversely correlated with the invasive potential of ovarian cancer cell lines and ovarian cancer specimens. We also demonstrated that DNA methylation in the *FILIP1L* promoter was a mechanism by which FILIP1L was down-regulated in ovarian cancer. In our present study, we tested this observation in other cancer histologies: breast, colon, lung and pancreatic cancers. Both mRNA and protein expression of FILIP1L were down-regulated in these cancer cells compared with their normal epithelial cells. As in ovarian cancer, DNA methylation is a mechanism by which FILIP1L is down-regulated in these cancer histologies. Methylation status of the *FILIP1L* promoter was inversely correlated with FILIP1L expression. Reduced methylation in the *FILIP1L* promoter following treatment with a DNA demethylating agent was associated with restoration of FILIP1L expression in these cancer cells. Further, FILIP1L expression was inversely correlated with the invasive potential of these cancer cells. Re-expression of FILIP1L in FILIP1L-low expressing, highly-invasive cancer cell lines resulted in inhibition of cell invasion. Correspondingly, knockdown of FILIP1L in FILIP1L-high expressing, low-invasive cancer cell lines resulted in increase of cell invasion. Overall, these findings suggest that down-regulation of FILIP1L associated with DNA methylation is related with the invasive phenotype in various cancers. Thus, modulation of FILIP1L expression has the potential to be a target for cancer therapy.

## Introduction

Cancer metastasis is the most common cause of cancer-related death, and invasive potential is correlated with poor outcomes in patients with a variety of cancers [1]. Characterization of the cellular mechanisms involved in cancer cell invasion and metastasis will allow for the development of more effective cancer therapies. We identified Filamin A interacting protein 1-like (FILIP1L; previously known as down-regulated in ovarian cancer 1 [DOC1]) as an important inhibitor of cell migration and invasion. Increased expression of FILIP1L resulted in inhibition of migration in endothelial cells [2] and inhibition of migration and invasion in cancer cells [3]. FILIP1L expression was inversely correlated with the invasive potential of ovarian cancer cell lines and ovarian cancer specimens [3]. Others have shown that intraperitoneal delivery of the *FILIP1L* gene resulted in inhibition of metastatic ovarian cancer spread into the peritoneum and intra-abdominal organs [4]. Overall, these findings suggest that FILIP1L may be an important inhibitor of cancer cell invasion and metastasis. 

To date, FILIP1L has been shown to be down-regulated only in ovarian and prostate cancers among human cancer histologies. *FILIP1L* mRNA was originally characterized by its presence in human ovarian surface epithelial (HOSE) cells and its absence in ovarian carcinoma cells [5]. *FILIP1L* down-regulation was confirmed by cDNA microarray analysis in ovarian carcinoma cells from patients with late-stage disease [6]. Differential gene expression analysis revealed that the *FILIP1L* gene in ovarian cancer cells presents several tagging single nucleotide polymorphisms [7]. *FILIP1L* was shown to be one of nine genes associated with functional suppression of tumorigenicity in ovarian cancer cell lines [8]. Using cDNA microarray analysis, *FILIP1L* was identified as one of the genes whose transcription is induced in senescent human prostate epithelial cells, but significantly repressed in immortalized prostate epithelial cells [9,10]. Recently, we and others have demonstrated that DNA methylation in the *FILIP1L* promoter was the mechanism by which FILIP1L was down-regulated in ovarian and prostate cancers [3,11].

Based on these observations, we asked whether FILIP1L expression was also down-regulated in other human cancer histologies and whether it was inversely correlated with the degree of invasive potential. In addition, since *FILIP1L* promoter methylation was associated with FILIP1L down-regulation in ovarian and prostate cancers [3,11], we examined whether or not the same mechanism is responsible for the down-regulation of FILIP1L in other cancer histologies.

Our results demonstrate that cellular invasion is inversely correlated with FILIP1L expression in human breast, colon, lung and pancreatic cancer cells. We observed that overexpression of FILIP1L inhibited the invasive potential of aggressive cancer cell lines of these histologies. We also demonstrate that *FILIP1L* promoter methylation is associated with FILIP1L down-regulation in these cancer cells. Taken together, these data suggest that the degree of FILIP1L expression may be a predictor of cancer cell behavior and, further, that the modulation of FILIP1L expression in various cancers may be a useful target for the development of novel cancer therapies.

## Materials and Methods

### Cell culture

All cell lines used were cultured in RPMI 1640 containing 10% fetal bovine serum (FBS), unless described separately. Human colon cancer cell lines HT-29, HCT 116, HCT-15, SW620 and T84 were purchased from the American Type Culture Collection (ATCC; Manassas, VA). The other human colon cancer cell lines Caco-2 and SW480, which were originally purchased from ATCC, were provided by Dr. Leonard Augenlicht, Albert Einstein College of Medicine, NY. Human lung cancer cell line H23 was purchased from ATCC. All the other human lung cancer cell lines – H322 and H1299, H460, A549 and H661, which were originally purchased from Sigma-Aldrich and ATCC, respectively – were provided by Dr. Roman Perez-Soler, Albert Einstein College of Medicine, NY. Human pancreatic cancer cell lines MIA PaCa-2, PANC-1, Hs 766T, HPAC, HPAF-II, SU.86.86, Panc 02.03 and Capan-1 and human breast cancer cell lines BT-549, Hs 578T, MDA-MB-468, BT-474 and ZR-75-1 were purchased from ATCC. The other human breast cancer cell lines MDA-MB-231 and MCF7, which were originally purchased from ATCC, were provided by Dr. Paraic Kenny, Albert Einstein College of Medicine, NY. Immortalized normal human colon cell line NCM460 was obtained from INCELL Corporation (San Antonio) and was cultured in M300F (INCELL) containing 10% FBS. Human primary cells human small airway epithelial cells (SAEC) and human mammary epithelial cells (HMEC) were purchased from Lonza and were cultured in SAGM and MEGM (Lonza), respectively.

### Quantitative real-time RT-PCR

Various cells, either untreated or DAC- and TSA-treated, were cultured and harvested at ~80% confluence. Total RNA was prepared by RNeasy kit (Qiagen), and cDNA was prepared by SuperScript VILO cDNA Synthesis Kit (Invitrogen). qRT-PCR was performed using a ViiA7 real-time PCR instrument as recommended by the manufacturer (Applied Biosystems). Expression of the *FILIP1L* gene was normalized to *hRPL7* gene expression. The TaqMan primers used were described previously [3].

### Western blot

Whole cell lysates were prepared from radioimmunoprecipitation assay (RIPA) buffer, separated on SDS-PAGE and transferred to nitrocellulose membrane. The membranes were blotted with antibodies against FILIP1L [2] and glyceraldehyde-3-phosphate dehydrogenase (GAPDH; Chemicon) followed by incubation with anti-mouse antibody conjugated to horseradish peroxidase. The signal was detected using chemiluminescence (Millipore). 

### Methylation analysis

Genomic DNAs from various cells were extracted using QIAamp DNA mini kit (Qiagen). Bisulphite modification was performed using EZ DNA Methylation kit (Zymo Research) following manufacturer's instructions. Bisulphite-modified DNA was subjected to nested PCR using HotStar Taq DNA Polymerase kit (Qiagen) following manufacturer's instructions. The nested PCR primers used were described previously [3]. Quantitative DNA methylation was analyzed by Sequenom® EpiTYPER Mass Array [12-15]. The assays were performed using the company's standard protocol through Genomics Shared Facility at Albert Einstein College of Medicine, NY. Matched peak data was exported using EpiTYPER software and analyzed quantitatively.

### 5-Aza-2’-deoxycytidine and Trichostatin A treatment

Various cancer cells were seeded in 6-well plates at a density of 1-2 × 10^5^ cells per well 16 h before treatment. Cells were treated with 5-aza-2’-deoxycytidine (DAC; Sigma-Aldrich) daily for 72 h or with Trichostatin A (TSA; Sigma-Aldrich) once for 24 h.

### Cell invasion assay

Various cells untransfected or transfected with wild-type *FILIP1L* or *FILIP1LΔC103* cDNA or *FILIP1L* siRNA were cultured at ~80% confluence. Cells were starved in basal medium containing 0.2% bovine serum albumin for 16 h. Matrigel invasion was measured using the BD BioCoat Tumor Invasion System (BD Biosciences #354165) as recommended by the manufacturer. Seeding 4.5 × 10^4^ of starved cells into the apical chambers was followed by adding a chemoattractant (10% FBS) to the basal chambers. After a 20 h incubation, quantification of cell invasion was achieved by post-cell invasion labeling with a fluorescent dye, calcein AM (BD Biosciences), and measuring the fluorescence of invading cells of the underside of the membrane at 494/517 nm (excitation/emission). Synergy Mx microplate reader (BioTek) was used to measure fluorescence, and Gen5 software (BioTek) was used to analyzed the data.

### Transfection of Cells with *FILIP1L* plasmids

Cloning of *FILIP1LΔC103* (amino acid 1-790) was described previously [2]. Plasmids were purified using Endo-free maxiprep kit (Qiagen). Various cancer cells were transfected with equimolar amounts of control empty plasmid or plasmid encoding wild-type *FILIP1L* or *FILIP1LΔC103* using X-fect solution following the manufacturer's protocols (Clontech). After a 24 h transfection, the cells were subjected to a cell invasion assay.

### Transfection of Cells with *FILIP1L* siRNA

ON-TARGETplus Non-Targeting siRNA Pool and SMARTpool of ON-TARGETplus *FILIP1L* siRNA was purchased from Thermo Scientific. Various cancer cells were transfected with equimolar amounts of either non-Targeting or *FILIP1L* siRNA using Dharmafect solution following the manufacturer's protocols (Thermo Scientific). After a 48 h transfection, the cells were subjected to a cell invasion assay.

### Statistical analysis

Statistical analyses were performed using a two-tailed Student's *t* test (GraphPad Prism 3.0), and differences were considered statistically significant at a value of *P* < 0.05. The correlation of *FILIP1L* mRNA expression with DNA methylation status of the CpG island in the *FILIP1L* promoter as well as with invasiveness of the cells was estimated by Spearman’s rank correlation method (GraphPad Prism 3.0).

## Results

### Differential expression of FILIP1L in various human cancer cell lines

**Figure 1:**
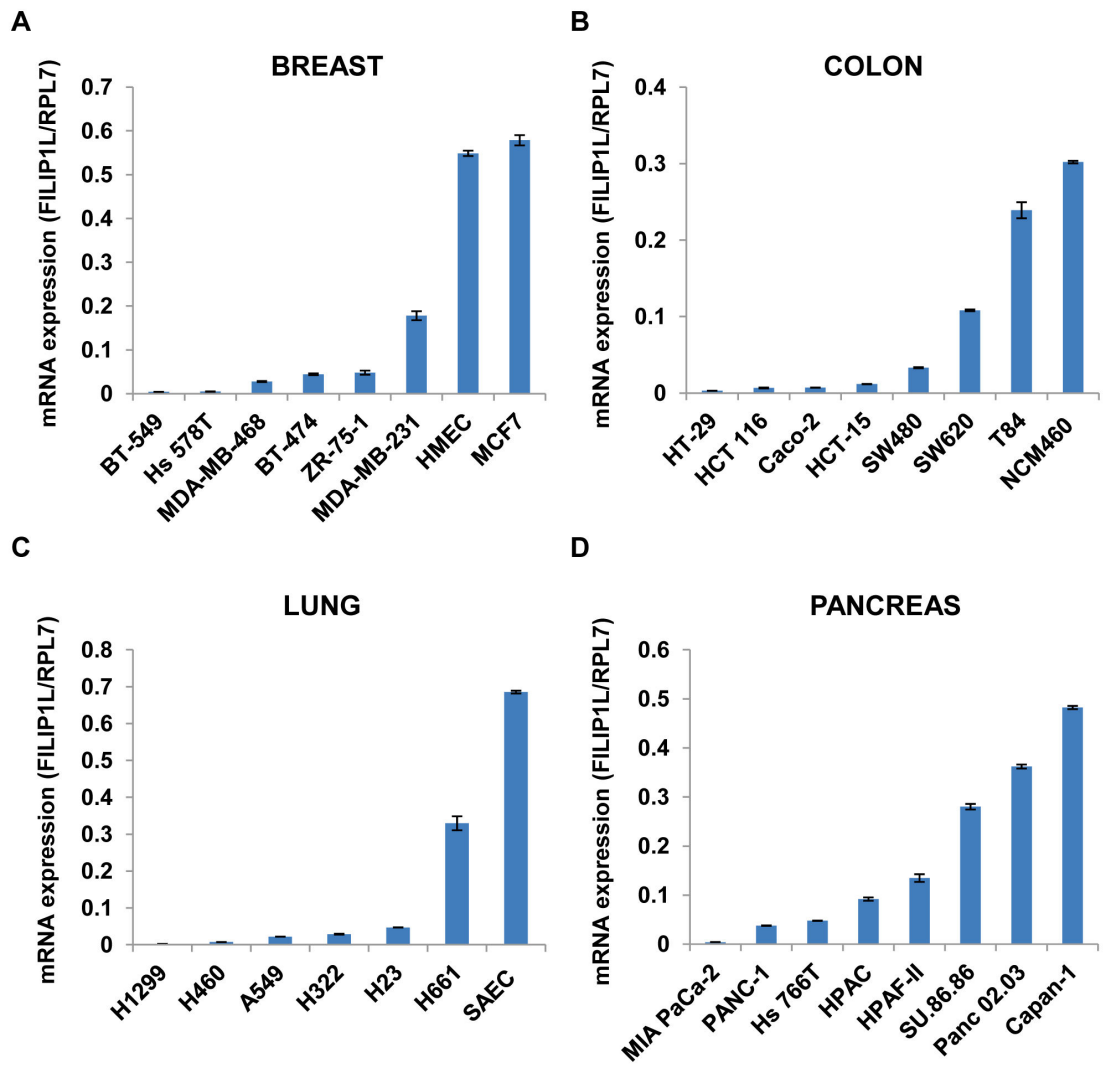
Differential expression of *FILIP1L* mRNA in various human cancer cell lines. qRT-PCR analysis for *FILIP1L* conducted on cDNA from human breast (*A*), colon (*B*), lung (*C*) and pancreatic (*D*) cancer cell lines. The *y* axis represents *FILIP1L* mRNA expression which was standardized with the housekeeping gene *hRPL7*. Error bars indicate SEM (*n* = 3). The result is an average of three independent experiments.

**Figure 2:**
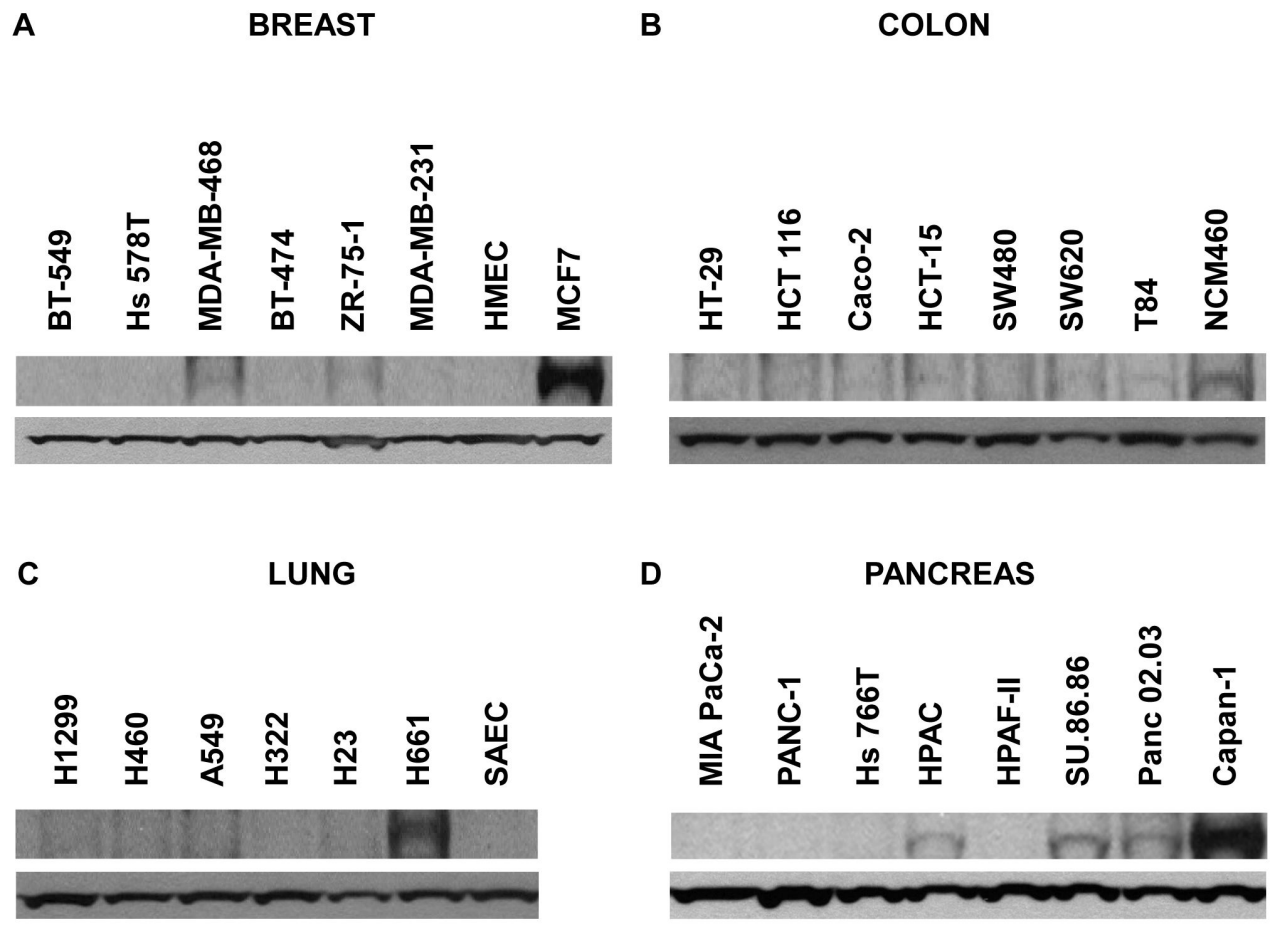
Differential expression of FILIP1L protein in various human cancer cell lines. Immunoblot analysis for FILIP1L in the same cells utilized in Figure 1. GAPDH blot is shown as the loading control. The result is representative of three independent experiments.

In order to test the expression levels of *FILIP1L* in various cancer cells, we measured *FILIP1L* mRNA expression in several human breast, colon, lung and pancreatic cancer cell lines by qRT-PCR. Normal human primary cells such as human small airway epithelial cells (SAEC) and human mammary epithelial cells (HMEC) as well as immortalized normal human colon cell line (NCM460) were also used. As shown in Figure 1A-C, mRNA expression of *FILIP1L* was highest in normal primary cells and cell line compared to cancer cell lines. For pancreatic cancer, normal cells were not included since they were not available. Interestingly, breast cancer cell line MCF7, which has been shown to be a non-invasive cell line [16], demonstrated as high *FILIP1L* expression as normal breast epithelial cells HMEC. In order to test if the protein expression of FILIP1L is correlated with its mRNA expression in these various cancer cells, we measured FILIP1L protein expression by immunoblot analysis using anti-FILIP1L antibody. As shown in Figure 2, FILIP1L protein expression was much higher in cells that express higher *FILIP1L* mRNA. FILIP1L protein was undetectable in many cancer cell lines. Interestingly, normal epithelial cells HMEC and SAEC did not show a robust FILIP1L protein band despite higher *FILIP1L* mRNA expression. These data demonstrate that the protein expression of FILIP1L is correlated with its mRNA expression in most cells from these histologies. Thus, these findings suggest that FILIP1L expression is down-regulated in various cancer cell lines compared with a normal cell line and non-invasive cancer cell lines.

### Inverse correlation of FILIP1L expression with the FILIP1L promoter methylation in various human cancer cell lines

**Figure 3:**
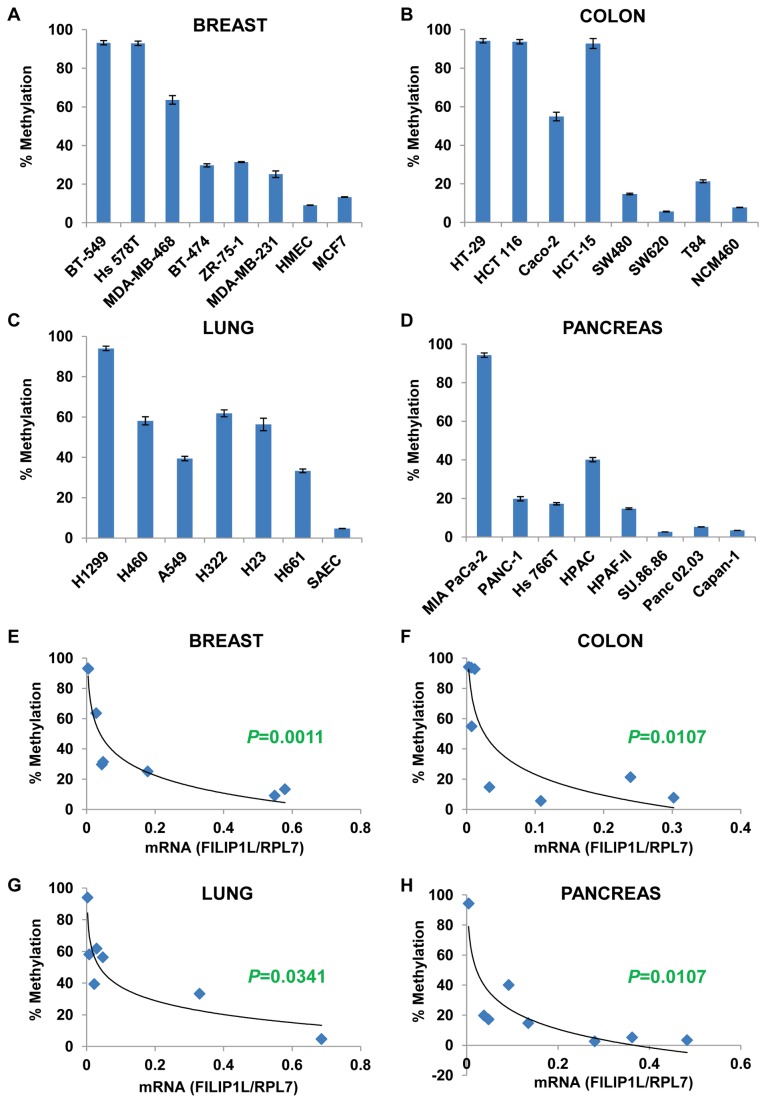
Inverse correlation of *FILIP1L* expression with *FILIP1L* promoter methylation in various human cancer cell lines. *A*-*D*, DNA methylation status of the CpG island in the *FILIP1L* promoter from the same cell lines utilized in Figure 1 was analyzed by Sequenom® EpiTYPER Mass Array. Mass Array results are shown as the average overall methylation for the analyzed 21 CG sites out of total 59 CG sites in the CpG island of the *FILIP1L* promoter. Error bars indicate SEM (*n* = 3). *E-H*, A significant inverse correlation of the DNA methylation status of the CpG island in the *FILIP1L* promoter with *FILIP1L* mRNA expression (each *P* value was calculated by Spearman’s rank correlation method). y axis: percent methylation of the average overall methylation for all 21 CG sites shown in sections *A-D* was used. x axis: standardized *FILIP1L* mRNA expression shown in Figure 1 was used.

We previously showed that the DNA methylation status at the *FILIP1L* promoter inversely correlated with FILIP1L expression [3]. As previously discussed, the average overall methylation was measured for the analyzed 21 CG sites out of total 59 CG sites in the CpG island of the *FILIP1L* promoter [3]. The average overall methylation demonstrated that these CG sites were highly methylated in cancer cells including BT-549 and Hs 578T breast cancer cells, HT-29, HCT 116 and HCT-15 colon cancer cells, H1299 lung cancer cells and MIA PaCa-2 pancreatic cancer cells (Figure 3A-D). In contrast, these sites were unmethylated in normal cells (HMEC, NCM460 and SAEC) as well as some cancer cell lines including MCF7 breast cancer cells, SW620 colon cancer cells and SU.86.86, Panc 02.03 and Capan-1 pancreatic cancer cells (Figure 3A-D). We tested if the DNA methylation status of the CpG island of the *FILIP1L* promoter (Figure 3A-D) inversely correlated with *FILIP1L* mRNA expression (Figure 1). As shown in Figure 3E-H, a significant inverse correlation was present in all four cancer histologies, suggesting that DNA methylation in the *FILIP1L* promoter may mediate FILIP1L down-regulation in various cancer histologies.

### Association of reduced methylation in the *FILIP1L* promoter with restoration of FILIP1L expression in various cancer cells following treatment with a DNA demethylating agent

**Figure 4:**
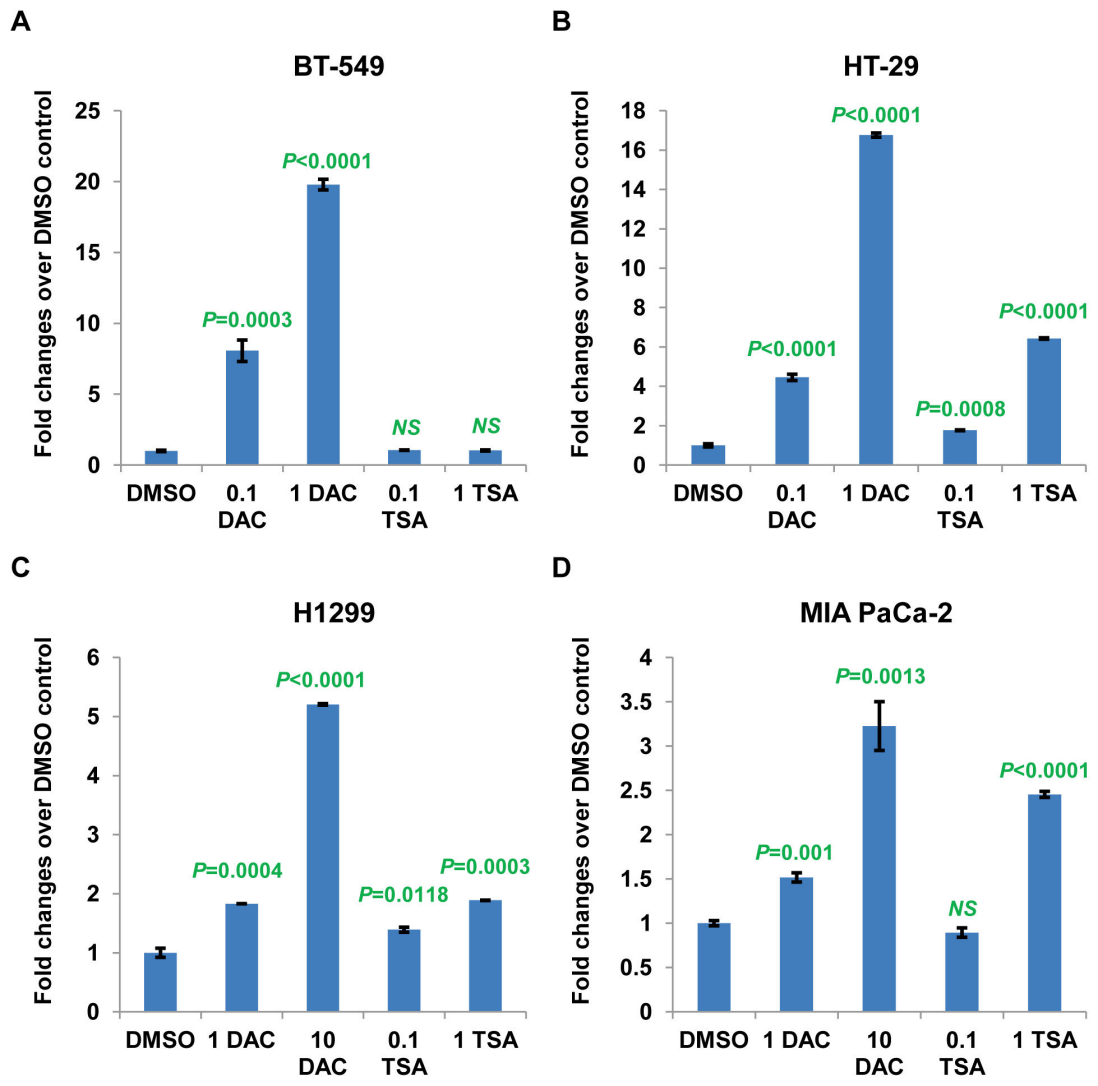
Restoration of *FILIP1L* expression in various cancer cells following treatment with a DNA demethylating agent or a histone deacetylase inhibitor. qRT-PCR analysis for *FILIP1L* conducted on cDNA from BT-549 breast (*A*), HT-29 colon (*B*), H1299 lung (*C*) and MIA PaCa-2 pancreatic (*D*) cancer cell lines treated with either 5-aza-2’-deoxycytidine (DAC) or Trichostatin A (TSA). Values 0.1, 1 or 10 in the *x* axis indicate the concentration of DAC and TSA in μM. The *y* axis represents fold change of each reagent-treated cell type over DMSO-treated control cells, where each value was standardized with the housekeeping gene *hRPL7*. Error bars indicate SEM (*n* = 3). The result is an average of two independent experiments. *P* values are derived from comparison between DMSO-treated control and either DAC- or TSA-treated experiments. NS indicates not significant.

**Figure 5:**
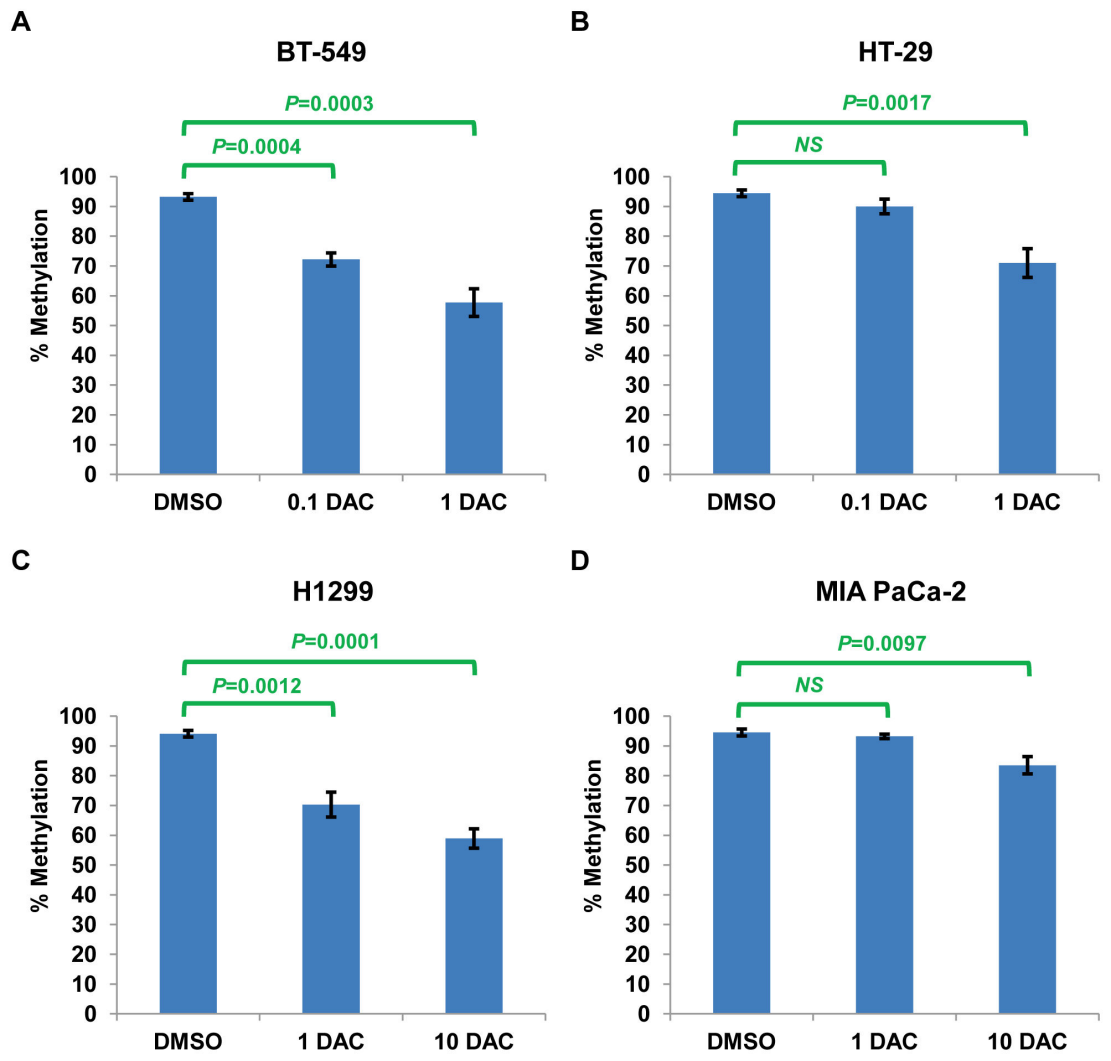
Association of reduced methylation in the *FILIP1L* promoter with restoration of FILIP1L expression in various cancer cells following treatment with a DNA demethylating agent. DNA methylation status of the CpG island in the *FILIP1L* promoter from the same cells used in Figure 4 was analyzed by Sequenom® EpiTYPER Mass Array. Mass Array results are shown as described in Figure 3A-D. The result is an average of two independent experiments. *P* values are derived from comparison between DMSO-treated control and each DAC-treated experiment.

To further test if epigenetic regulation results in re-expression of FILIP1L, we treated the four lowestFILIP1L-expressing cancer cell lines from each cancer histology (BT-549, HT-29, H1299 and MIA PaCa-2; Figure 1) with either a DNA demethylating agent or histone deacetylase inhibitor. Treatment of these cells with DNA demethylating agent 5-aza-2’-deoxycytidine (DAC) resulted in significantly increased *FILIP1L* mRNA expression in all four cancer histologies (Figure 4). Interestingly, histone deacetylase inhibitor Trichostatin A (TSA) treatment also resulted in significantly increased *FILIP1L* mRNA expression in these cancer cell lines, except for the BT-549 breast cancer cell line. *FILIP1L* mRNA expression in DAC- or TSA-treated cells was compared with that in DMSO control-treated cells. In order to examine whether reduced methylation in the *FILIP1L* promoter is associated with restoration of FILIP1L expression, we analyzed the methylation status of the *FILIP1L* promoter in the cells utilized in Figure 4 following DAC treatment. Most CG sites analyzed in the CpG island of the *FILIP1L* promoter demonstrated similar reduction in percent methylation in each cell type (data not shown). As shown in Figure 5, all four cell lines treated with DAC demonstrated a significant decrease in the average overall methylation. These data demonstrate that DNA methylation in the *FILIP1L* promoter is a mechanism by which FILIP1L is down-regulated in various cancer histologies.

### Inverse correlation of FILIP1L expression with the invasive potential of various human cancer cell lines

**Figure 6:**
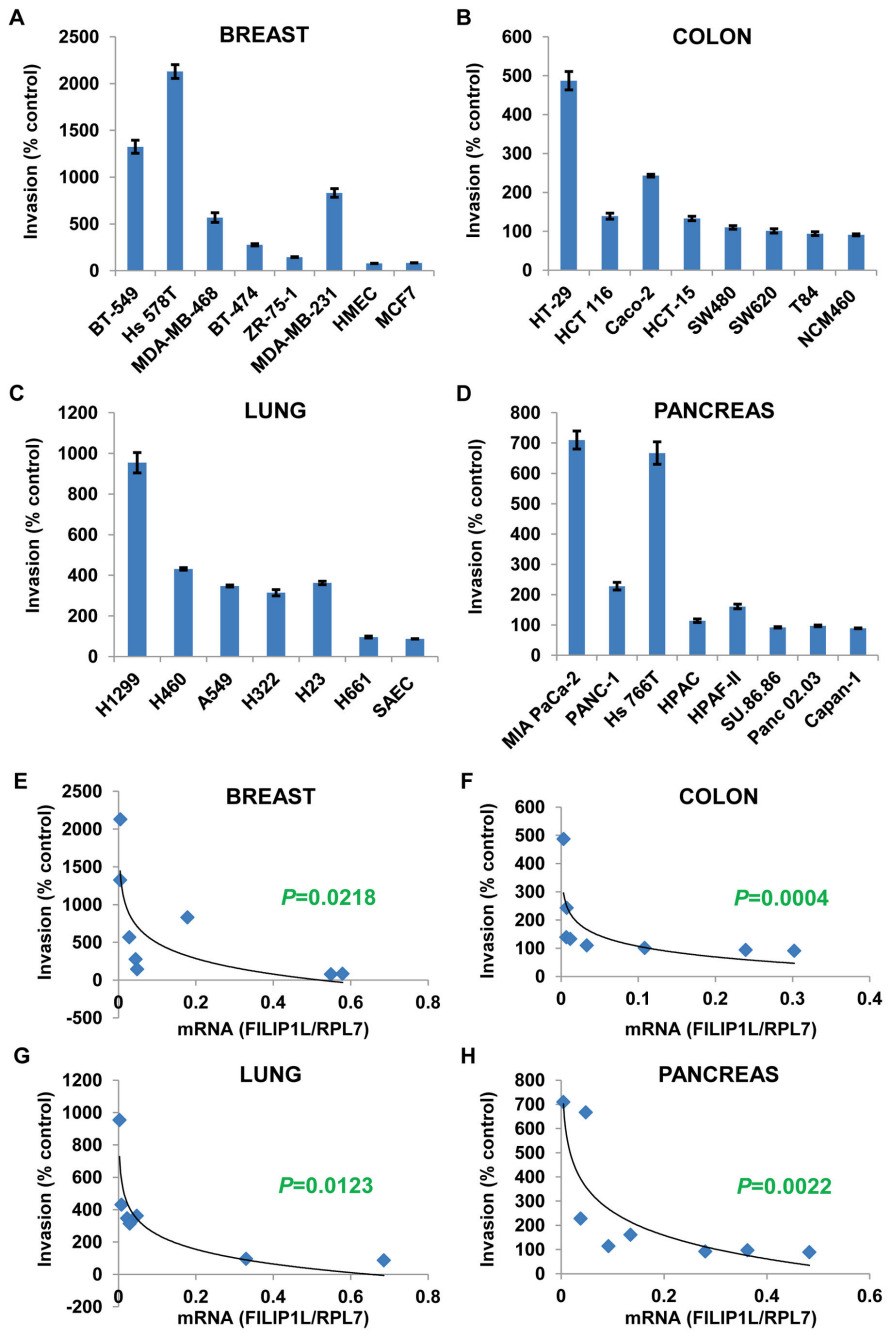
Inverse correlation of FILIP1L expression with the invasive potential of various human cancer cell lines. *A*-*D*, Matrigel cell invasion assay for the same cells utilized in Figure 1. Matrigel invasion was measured using the BD BioCoat Tumor Invasion System as described in *Materials and Methods*. The *y* axis represents a percent change over serum-free control. Error bars indicate SEM (*n* = 4). The result is representative of three independent experiments. *E-H*, A significant inverse correlation of the *FILIP1L* mRNA expression with invasiveness of the cells (each *P* value calculated by Spearman’s rank correlation method). y axis: invasiveness of the cells as a percent change over serum-free control shown in sections *A-D* was used. x axis: standardized *FILIP1L* mRNA expression shown in Figure 1 was used.

**Figure 7:**
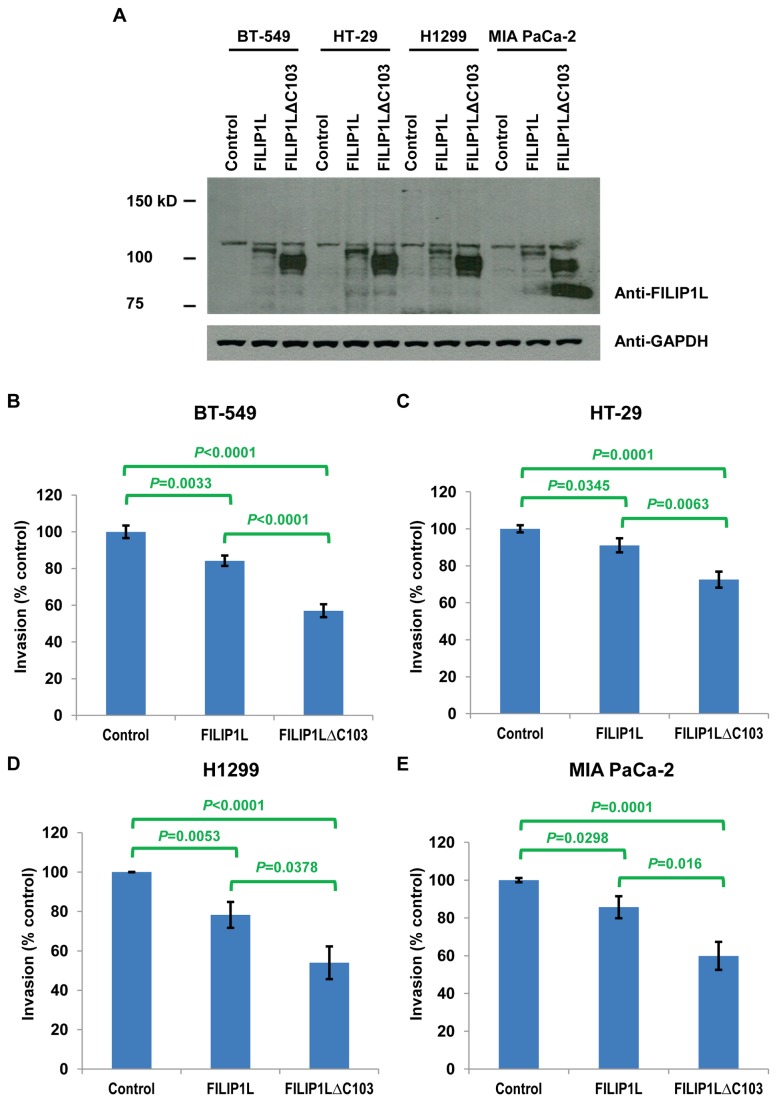
Inhibition of cell invasion following FILIP1L expression in various FILIP1L-low-expressing, highly-invasive cancer cell lines. The same cell lines used in Figure 4 were transfected with control, wild-type *FILIP1L* or *FILIP1LΔC103* cDNA. *A*, Immunoblot analysis for FILIP1L in the transfected cells. GAPDH blot is shown as the loading control. *B*-*E*, Transfected cells were subject to Matrigel cell invasion assay 24 h after transfection. The same experimental procedures were followed as described in Figure 6. The result is representative of two independent experiments.

**Figure 8:**
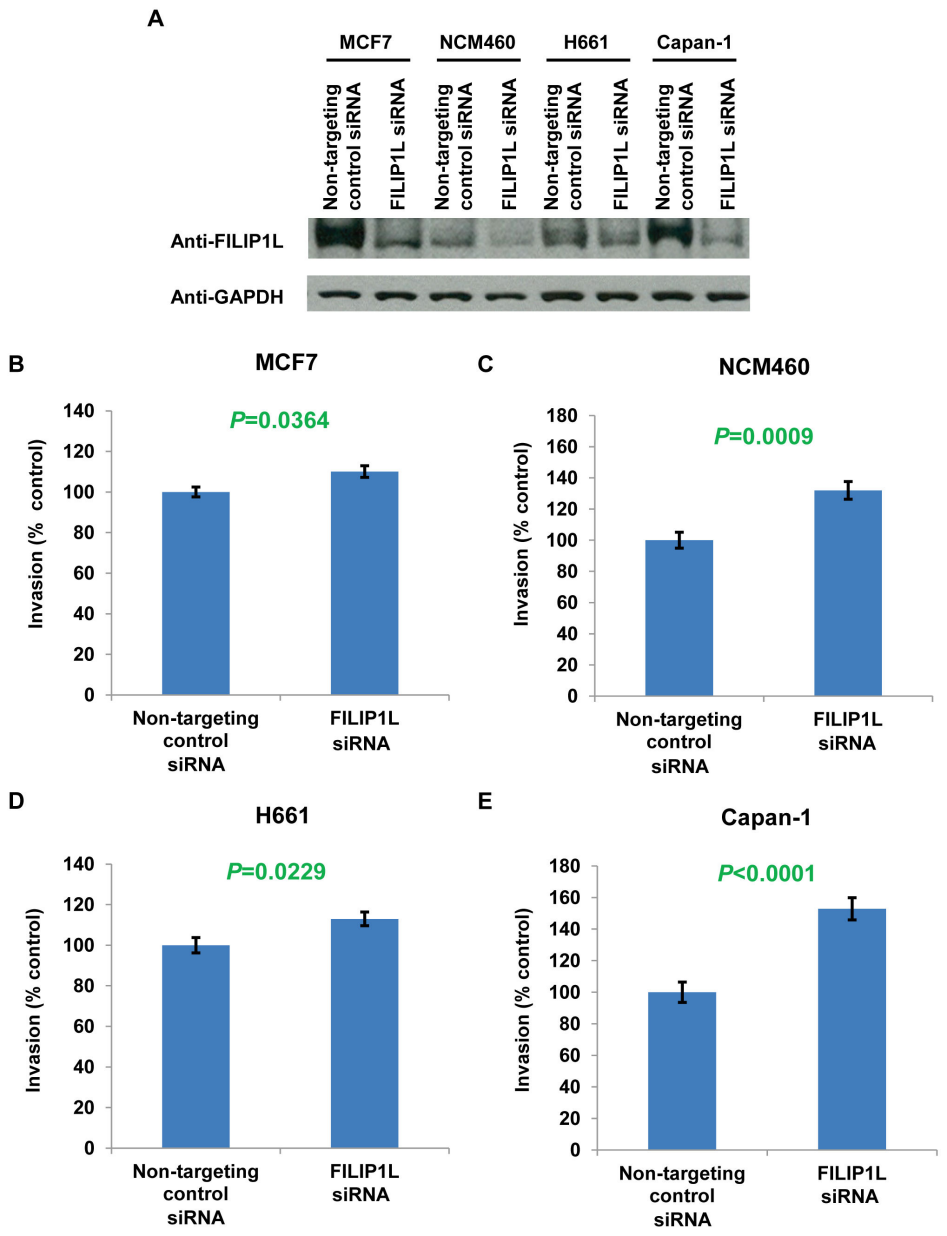
Increase of cell invasion following FILIP1L knockdown in various FILIP1L-high-expressing, low-invasive cancer cell lines. MCF7 breast (*B*), NCM460 colon (*C*), H661 lung (*D*) and Capan-1 pancreatic (*E*) cancer cell lines were transfected with either non-targeting or *FILIP1L* siRNA. *A*, Immunoblot analysis for FILIP1L in the transfected cells. GAPDH blot is shown as the loading control. *B*-*E*, Transfected cells were subject to Matrigel cell invasion assay 48 h after transfection. The same experimental procedures were followed as described in Figure 6. The result is representative of two independent experiments.

We previously demonstrated that the down-regulation of FILIP1L expression associated with DNA methylation in the *FILIP1L* promoter is related with invasive potential in ovarian cancer cell lines [3]. We tested whether *FILIP1L* expression is inversely correlated with the invasive potential of breast, colon, lung and pancreatic cancer cell lines. We examined the invasive activity for the same cell lines utilized in Figure 1 by Matrigel invasion assay. As shown in Figure 6A-D, most of the cell lines that demonstrated low *FILIP1L* expression invaded Matrigel significantly more than those that demonstrated high *FILIP1L* expression. In addition, the *FILIP1L* mRNA expression demonstrated a significant inverse correlation with invasiveness of the cells (Figure 6E-H), suggesting that *FILIP1L* expression is inversely correlated with the invasive potential of all four cancer cell lines. We then tested if overexpression of FILIP1L in FILIP1L-low-expressing, highly-invasive cancer cell lines resulted in inhibition of cell invasion. The same cell lines utilized in Figure 4 and 5 were tested. Our previous studies have shown that overexpression of FILIP1LΔC103 (COOH terminal truncation mutant 1-790) as well as wild-type FILIP1L resulted in inhibition of ovarian cancer cell invasion [3]. Thus, we transfected these various cancer cells with a plasmid encoding control, wild-type *FILIP1L* or *FILIP1LΔC103* cDNA, and measured invasion. Under the same experimental condition, the expression level of FILIP1LΔC103 was higher than that of wild-type FILIP1L in all four cell lines (Figure 7A). As shown in Figure 7B-E, all four cell lines transfected with either wild-type *FILIP1L* or *FILIP1LΔC103* cDNA invaded Matrigel significantly less than those transfected with control. To definitively prove that FILIP1L inversely regulates invasive properties of the cancer cells, we tested if knockdown of FILIP1L in FILIP1L-high expressing, low-invasive cancer cell lines resulted in an increase in cell invasion. We transfected these various cancer cells with either non-targeting or *FILIP1L* siRNA and measured invasion. As shown in Figure 8A, all four cell lines transfected with *FILIP1L* siRNA demonstrated a reduction of FILIP1L compared with control. All four cell lines transfected with *FILIP1L* siRNA invaded Matrigel significantly more than those transfected with control siRNA (Figure 8B-E). Collectively, these data suggest that down-regulation of FILIP1L is associated with an invasive phenotype in various cancer cell lines and that this phenotype can be reversed by overexpression of FILIP1L.

## Discussion

Cancer cell invasion is a critical first step in cancer metastasis, and the invasive potential of cancer cells is correlated with poor outcomes in patients with a variety of cancers [1]. Characterization of the cellular mechanisms involved in invasion will allow for the development of more effective cancer therapies. We previously demonstrated that FILIP1L expression was inversely correlated with the invasive potential of ovarian cancer cell lines and ovarian cancer specimens [3]. In the present study, we have shown that FILIP1L expression is inversely correlated with the invasive potential of various cancer cell lines such as breast, colon, lung and pancreatic cancer. Others have shown that FILIP1L expression was down-regulated in prostate cancers compared with normal tissues [11]. Thus, findings from the present study suggest that FILIP1L could be down-regulated and inversely correlated with the invasive potential of many human cancer histologies. Furthermore, it was recently shown that intraperitoneal delivery of the *FILIP1L* gene resulted in inhibition of metastatic ovarian cancer spread into the peritoneum and intra-abdominal organs [4]. Overall, these findings suggest that FILIP1L may be an important inhibitor of cancer cell invasion and metastasis in a wide variety of tumor types. Therefore, it will be of interest to identify the mechanism by which FILIP1L inhibits cancer cell invasion and metastasis in these various cancer histologies.

We initially identified FILIP1L in the setting of tumor angiogenesis. In order to identify common intracellular mediators of proliferation, migration and apoptosis in endothelial cells, we previously analyzed gene expression profiles of endothelial cells after treatment with angiogenesis inhibitors such as endostatin, fumagillin and EMAP-II [17,18]. *FILIP1L* was up-regulated in endothelial cells in response to these inhibitors. We subsequently demonstrated that overexpression of FILIP1L resulted in inhibition of cell proliferation and migration and increased apoptosis in endothelial cells [2]. In addition, targeted expression of FILIP1L in the tumor vasculature inhibited tumor growth *in vivo* [2]. These findings suggested that FILIP1L may be an important inhibitor of cell proliferation and migration as well as an inducer of apoptosis in endothelial cells. Along with the findings from the present study, it suggests that FILIP1L may be utilized as a cancer therapeutic, which can inhibit cancer metastasis as well as angiogenesis.

Normal epithelial cells HMEC and SAEC did not show FILIP1L protein expression despite higher *FILIP1L* mRNA expression. When we previously performed the same experiments using normal ovarian epithelial cells, the protein level strongly correlated with mRNA level [3]. This discrepancy could be explained by posttranscriptional mRNA degradation, translational efficiency and/or protein degradation, which have been shown to be dynamic and cell specific [19-24]. Currently, we do not have an explanation for the lack of FILIP1L protein detection, and we are exploring this phenomenon further. Understanding a cell-type specific regulation of FILIP1L protein expression may give us insights into the regulation of this anti-invasive protein.

FILIP1LΔC103, a COOH terminal truncation mutant of FILIP1L demonstrated a greater anti-invasion activity than the wild-type FILIP1L. The wild-type FILIP1L has 893 amino acids. As previously described [2], the NH2 terminal half of the FILIP1L protein consists of a coiled-coil region (residues 3-542), two leucine zipper motifs (residues 83-111 and 218-253) and a prefoldin domain (residues 465-535), and has a SbcC (COG0419; ATPase involved in DNA repair; residues 19-576) conserved domain. Its COOH terminal half is an unstructured region and has a Herpes_BLLF1 (pfam05109; Herpes virus major outer envelope glycoprotein; residues 640-829) conserved domain. As shown in Figure 7A, the expression level of FILIP1LΔC103 was greater than that of wild-type FILIP1L, which may explain the observation of a greater anti-invasive activity of FILIP1LΔC103 compared to wild-type FILIP1L. The same results were observed when ovarian cancer cells were tested previously [3]. Deleting 103 amino acids from the COOH terminus of FILIP1L greatly enhanced protein stability in cancer cells. Whether disrupting a Herpes_BLLF1 conserved domain is directly involved in enhancing FILIP1L’s stability is not currently known.

We previously showed that FILIP1L expression was significantly lower in invasive serous carcinoma than in non-invasive serous borderline tumors [3]. The methylation of the CpG island in the *FILIP1L* promoter was significantly higher in invasive serous carcinomas than that in non-invasive serous borderline tumors [3]. Others have shown that FILIP1L expression was significantly lower and that *FILIP1L* promoter methylation was significantly higher in prostate carcinoma samples compared with matched normal tissues [11]. Thus, we believe it would be important to confirm if FILIP1L is down-regulated in tissues of other cancer histologies such as breast, colon, lung and pancreas, and whether or not *FILIP1L* promoter methylation is a mechanism by which FILIP1L is down-regulated in these cancer tissues.

We have shown that a DNA demethylating agent DAC, but not the histone deacetylase inhibitor TSA, resulted in increased *FILIP1L* mRNA in FILIP1L-low-expressing ovarian [3] and breast (Figure 4A) cancer cells, suggesting that DNA methylation, but not histone modification, is associated with the down-regulation of FILIP1L in these cells. However, TSA treatment also resulted in increased *FILIP1L* mRNA in cancer cells from other histologies such as colon, lung and pancreas (Figure 4B-D), which suggests that DNA methylation is not the only mechanism by which FILIP1L is down-regulated in these cells. It will be necessary to further examine whether there is an additive effect of DNA methylation and histone modification on FILIP1L down-regulation. In addition, examination of the potential preference of either or both mechanisms by cell- and/or tissue-specificity will also improve the understanding of how FILIP1L is down-regulated.
