# Correction: Down-Regulation of Filamin Ainteracting protein 1-like Is Associated with Promoter Methylation and an Invasive Phenotype in Breast, Colon, Lung and Pancreatic Cancers

**DOI:** 10.1371/annotation/b2ee22c9-aa14-4c03-861c-b37f1a842bcd

**Published:** 2013-12-30

**Authors:** Mijung Kwon, Soo Jin Lee, Srilakshmi Reddy, Yevangelina Rybak, Asha Adem, Steven K. Libutti

The publisher apologizes for the following typesetting errors. A space was erroneously omitted from the title. The correct title should read: 

"Down-Regulation of Filamin A interacting protein 1-like Is Associated with Promoter Methylation and an Invasive Phenotype in Breast, Colon, Lung and Pancreatic Cancers."

There was an error in the Discussion section. The final paragraph was erroneously placed in the Acknowledgments. The final paragraph of the Discussion should be: 

"In summary, we have demonstrated that down-regulation of FILIP1L is associated with the invasive phenotype in cancer cells of various histologies. FILIP1L promoter methylation is associated with down-regulation of FILIP1L in these cancer cells. Further characterization of the mechanism of FILIP1L down-regulation may provide us with the understanding of the role played by FILIP1L in carcinogenesis and lead to the development of more effective anticancer therapies."

